# Associations of Age With Tumor Genomic Characteristics in Relapsed/Refractory Cancers Interrogated With the NCI-MATCH Trial Targeted Gene Panel Assay

**DOI:** 10.1200/PO-25-01183

**Published:** 2026-08-07

**Authors:** Hari Sankaran, Yuri Kotliarov, Yingdong Zhao, Lyndsay N. Harris, James V. Tricoli, Stanley R. Hamilton, Sarah M. Temkin, Chris Karlovich, Ting-Chia Chang, Victoria Wang, Robert J. Gray, Zihan Wei, Sarah J. Shin, Nita L. Seibel, Biswajit Das, Brent Coffey, David Patton, Ming-Chung Li, Jessica Li, Ana F. Best, P. Mickey Williams, A. John Iafrate, Jeffrey Sklar, Keith T. Flaherty, Alice P. Chen, Peter J. O'Dwyer, Lisa M. McShane

**Affiliations:** ^1^ Biometric Research Program, Division of Cancer Treatment and Diagnosis, National Cancer Institute, Bethesda, MD; ^2^ Cancer Diagnosis Program, Division of Cancer Treatment and Diagnosis, National Cancer Institute, Bethesda, MD; ^3^ Division of Cancer Treatment and Diagnosis, National Cancer Institute, Bethesda, MD; ^4^ City of Hope Comprehensive Cancer Center, Duarte, CA; ^5^ Office of Research on Women's Health, NIH, Bethesda, MD; ^6^ Molecular Characterization Laboratory, Frederick National Laboratory for Cancer Research, Frederick, MD; ^7^ Dana Farber Cancer Institute-ECOG-ACRIN Biostatistical Center, Boston, MA; ^8^ Division of Cancer Treatment and Diagnosis, Developmental Therapeutics Clinic, National Cancer Institute, Bethesda, MD; ^9^ Division of Cancer Treatment and Diagnosis, Clinical Investigations Branch, National Cancer Institute, Bethesda, MD; ^10^ Center for Biomedical Informatics and Information Technology, National Cancer Institute, Bethesda, MD; ^11^ Pathology Department, Massachusetts General Hospital, Boston, MA; ^12^ Yale University, New Haven, CT; ^13^ Massachusetts General Hospital Cancer Center, Boston, MA; ^14^ University of Pennsylvania, Philadelphia, PA

## Abstract

**PURPOSE:**

Motivated by the rising incidence of cancers at younger ages, this study compares tumor genomic alterations between adolescents and young adults (AYAs; 18-39 years) and non-AYAs (40 years and older) and explores the relationship with continuous age in patients with relapsed/refractory ovarian, breast, and colorectal cancers accrued to the NCI-MATCH trial.

**METHODS:**

Tumor genomic profiles generated by a next-generation sequencing 143-gene panel (NCI-MATCH assay, v2) were analyzed for association with age (AYA/total: 21/455 ovarian, 27/576 breast, 43/759 colorectal cancers). For each gene, AYA and non-AYA DNA alteration proportions were compared (Fisher exact test) and alteration association with continuous age (logistic regression) was evaluated (false discovery rate‑adjusted *P* value <.1 statistically significant). For colorectal cancer, sex-stratified analysis was also performed.

**RESULTS:**

No significant AYA versus non-AYA differences were observed in the prevalence of gene mutations (single nucleotide variant [SNV]/indel). A significant association of gene amplification with AYAs (odds ratio [OR], 95% CI) was *CCND1* (0.2, 0.1-0.4), favoring AYAs in breast cancer. Examining age as a continuous variable, significant associations of gene mutations (SNV/indel) with older age, expressed as 5-year OR (OR [95% CI]), were observed: *TP53* (1.3 [1.1 to 1.4]), ovarian cancer; *CDH1* (1.4 [1.2 to 1.6]) and *PIK3CA* (1.1 [1.1 to 1.2]), breast cancer; and *BRAF* (1.4 [1.2 to 1.7]), female colorectal cancer. Associations with younger age included *SMAD4* (0.8 [0.7 to 0.9]), male colorectal cancer. Significant associations of gene amplification with age (continuous) were as follows: *CCNE1* (1.3 [1.1 to 1.6]), older ovarian cancer, and *CCND1* (0.8 [0.8 to 0.9]), younger breast cancer.

**CONCLUSION:**

Comparing AYAs with non-AYAs among patients having relapsed/refractory disease, no significant differences in SNV/indel prevalence were observed, but *CCND1* amplifications were more prevalent in AYA breast cancer. For several genes, DNA alterations were associated with continuous age and may depend on sex in colorectal cancer.

## INTRODUCTION

The Molecular Analysis for Therapy Choice (*NCI-MATCH*, NCT02465060) trial is a precision oncology platform trial that ultimately included 38 single-arm phase II substudies evaluating activity of molecularly targeted anticancer agents in patients aged 18 years and older with any advanced solid tumor, including CNS malignancies, lymphoma, or myeloma, who progressed on standard treatment or for whom no standard treatment was available.^1^ Tumor genomic profiles of nearly 6,000 patients in NCI-MATCH using a rigorous, standardized sequencing procedure provide a rich resource for investigating several questions concerning biology of advanced cancers. Motivated by accumulating evidence of increasing incidence of certain cancers in the adolescent and young adult (AYA) age group (conventionally defined as 15-39 years in cancer),^2-4^ this study examined whether tumor genomic profiles seemed to differ between AYA and older adults or changed according to age on a continuum. This report is limited to three cancer types enrolling sufficient numbers of AYA patients (restricted to age 18 years and older) to permit robust analyses: ovarian, breast, and colorectal cancers. Results presented herein provide further insights into the question of whether cancers among AYAs have substantially different genomic profiles than in older adults or merely represent earlier manifestation of fundamentally the same diseases.

**CONTEXT**

**Key Objective**
Are there genomic differences related to age in relapsed/refractory cancers?
**Knowledge Generated**
Tumor genomic profiles assessed by the NCI-MATCH clinical assay were generally similar between adolescent and young adult (AYA) and older patients. Associations with age analyzed as a continuous variable were observed in ovarian (*TP53* and *CCNE1*), breast (*CDH1*, *PIK3CA*, and *CCND1*), and colorectal (*BRAF* and *SMAD4*) cancers stratified by sex.
**Relevance**
Findings in this study on the basis of the NCI-MATCH assay revealed associations with age, but they do not suggest different tumor biology that would translate to alternative therapeutic recommendations specifically for AYA patients.

### Ovarian Cancer

Most ovarian cancers (approximately 90%) are fallopian tube in origin; however, malignancies also arise from germ cells and stromal tissue.^5^ Germ cell tumors are more common in the pediatric age range. Stromal tumors are diagnosed across all age groups. Incidence of epithelial ovarian cancers, especially high-grade serous carcinoma (HGSC), increases with age. Ovarian cancers occurring in AYA patients tend to be lower grade, lower stage, and less aggressive malignancies.^6^

Complexity of the genomic landscape across ovarian cancer histotypes, within subtypes, between patients and from different metastatic locations within the same patient,^7,8^ has resulted in challenges in identifying effective targeted therapies.^9-11^ Mutations in homologous recombination genes (eg, *BRCA1* and *BRCA2*) are identified in up to one third of ovarian carcinomas, most commonly in high-grade serous ones. *TP53* mutations, including in-frame or frameshift insertions and deletions and missense and nonsense mutations, are a distinguishing characteristic of HGSC.^5,11^ Clear cell and endometrioid carcinomas are associated with *ARID1A* and *CTNNB1* mutations.^8,12^ Mutations in the MAPK pathway are common in low-grade serous and mucinous carcinomas and are the most commonly identified actionable mutation in tumors occurring in AYA patients.^13^

### Breast Cancer

Breast cancer (BRC) in young women (younger than 40 years) has consistently been shown to be associated with worse prognosis^14^ overall, with higher likelihood of local recurrence and development of contralateral BRC. Most BRCs are invasive ductal carcinoma (approximately 75%), then invasive lobular (approximately 15%), and finally rare subtypes including mucinous, medullary, papillary, and tubular (approximately 10%)^15^; histopathologic features more common in young women, including higher tumor grade and increase in number of positive lymph nodes, may partially account for worse clinical outcome.^16,17^ Human epidermal growth factor receptor 2 (HER2)-positive and estrogen receptor (ER) and progesterone receptor (PgR)-negative tumors are more common in women younger than 40 years and are associated with more aggressive disease, but this association does not fully explain worse outcomes in this age group.^18,19^ Other genes/pathways reported altered more frequently in tumors of women younger than 45 years compared with older have included *EGFR*, *TP53*, *GATA3*, *ARIDIA*, and the PI3K pathway; further studies have suggested that *BRCA1* mutation and a related signature may be associated with BRC in younger women. Gene expression studies have reported several pathways including PI3K, mammary stem cell, and luminal progenitor pathways to be enriched in women aged 45 years or younger when compared with women older than 45 years.^20,21^

### Colorectal Cancer

Most colorectal cancers (CRCs) arise from the epithelium through a complex series of mutational events in the genes *APC*, *KRAS*, *DCC*, *TP53*, and others that can take place over a long period of time^22^ or as a result of inherited mutations, such as in Lynch syndrome.^23^ Despite the overall decrease in CRC incidence and mortality over the past several decades, younger individuals continue to demonstrate opposite trends in both incidence and mortality.^24-26^ Incidence of CRC in AYAs has risen substantially in this period, with an annual increase of 2.6% since 1988.^27^ This trend is particularly concerning given worse outcomes seen in the AYA population.^28^ Previous studies have found differences in mutation profiles between AYA, adult, and pediatric cohorts.^29-31^ A recent review describes sex-based differences in the biology of CRC and patient outcomes; these may be related to sex hormone levels, which also vary with age.^32^

## METHODS

Patients initially considered for inclusion in this ancillary study of NCI-MATCH were those accrued during the trial's central screening phase (N = 5,961): 321 overlapped the AYA age range (18-39 years) and 5,640 were older than 39 years. Select clinical, demographic, immunohistochemistry, and other assay data were provided from the trial database.

**FIG 1.**
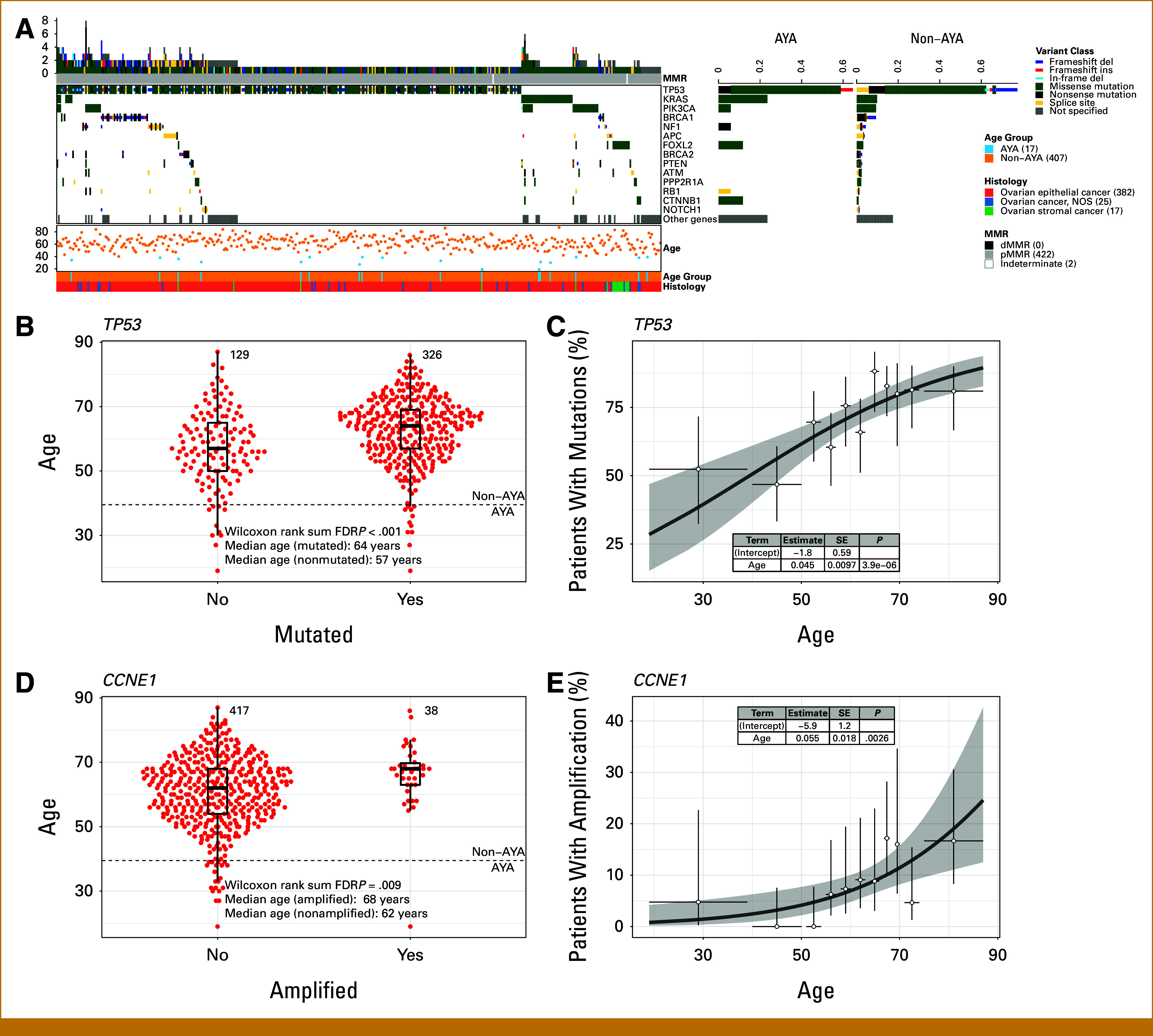
(A) The genomic landscape (SNV/indels) of 424 (17 AYA) patients with ovarian cancer screened on the NCI-MATCH trial. Only patients with at least 1 mutation are shown, excluding 31 patients. Each column in the oncoprint represents a patient, and each row represents a mutated gene. Fourteen genes were mutated in more than five patients. Other genes mutated in five or fewer patients are combined in a single row and colored in gray. If different types of mutations (eg, frame shift deletion and missense mutation in TP53) were present in the same patient, they are represented with their respective colors. The stacked bar plot above the oncoprint is the number of different types of mutations per patient overall. The stacked horizontal bars to the right of the oncoprint display the proportion of mutations in AYA and non-AYA patients for each selected gene. (B-E) Association of age with selected alterations in ovarian cancer. (B) Boxplot showing distribution of ages of patients with and without *TP53* mutation. The division of AYAs and non-AYAs is demarcated by a dashed line at 40 years. (C) Relationship of probability of *TP53* mutation with patient age along with a fitted logistic regression model. Fitted model parameters are shown in the insert table. Observed proportion mutated with 95% CIs (vertical bars) are displayed for age categories 18-39 years (AYA) and by decile of age starting at 40 years, with horizontal bar indicating age bins. (D) Boxplot showing distribution of ages of patients with and without *CCNE1* amplification (similar to B). (E) Relationship of probability of *CCNE1* amplification with patient age along with a fitted logistic regression model (similar to C). AYA, adolescent and young adult; dMMR, deficient mismatch repair; FDRp, false discovery rate‑adjusted *P* value; NOS, not otherwise specified; pMMR, proficient mismatch repair; SNV, single nucleotide variant.

Patients were centrally screened for NCI-MATCH using the next-generation sequencing assay (referred to here as the NCI-MATCH assay) on the basis of the Oncomine Comprehensive Assay from ThermoFisher Scientific.^33^ As most of the patients (88% of 5,533 successfully assayed patients) in NCI-MATCH were screened using the NCI-MATCH v2 assay,^34^ analyses were restricted to those patients to ensure comparability. Additional details are described in the Supplement (Fig 1 and Table 1).

**TABLE 1.** Demographic Characteristics of Cohort

Variable	Ovarian (n = 455)		Breast (n = 576)		Colorectal (n = 759)	
	AYA (n = 21)	Non-AYA (n = 434)	AYA (n = 27)	Non-AYA (n = 549)	AYA (n = 43)	Non-AYA (n = 716)
Median age at enrollment (range)	63 years (19-87)		59 years (24-87)		60 years (26-89)	
	33 (19-39)	63 (40-87)	36 (24-39)	60 (40-87)	36 (26-29)	60.5 (40-89)
Sex, n (%)						
Male	—	—	0 (0.0)	7 (1.3)	19 (44.2)	392 (54.7)
Female	21 (100.0)	434 (100.0)	27 (100.0)	542 (98.7)	24 (55.8)	324 (45.3)
Race, n (%)						
White	14 (66.7)	357 (82.3)	18 (66.7)	447 (81.4)	31 (72.1)	568 (79.3)
Black or African American	2 (9.5)	26 (6.0)	7 (25.9)	56 (10.2)	6 (14.0)	67 (9.4)
Asian	3 (14.3)	22 (5.1)	0 (0)	18 (3.3)	2 (4.7)	31 (4.3)
Other^a^	0 (0)	2 (0.5)	0 (0)	9 (1.6)	1 (2.3)	10 (1.4)
Not available	2 (9.5)	27 (6.2)	2 (7.4)	19 (3.5)	3 (7.0)	40 (5.6)
Ethnicity, n (%)						
Not Hispanic or Latino	19 (90.5)	397 (91.5)	25 (92.6)	493 (89.8)	37 (86.0)	651 (90.9)
Hispanic or Latino	1 (4.8)	14 (3.2)	1 (3.7)	34 (6.2)	4 (9.3)	45 (6.3)
Not available	1 (4.8)	23 (5.3)	1 (3.7)	22 (4.0)	2 (4.7)	20 (2.8)

Abbreviation: AYA, adolescent and young adult.

^a^ Other includes Native Hawaiian or Other Pacific Islander, American Indian or Alaska Native, and Multirace.

Analyses focused on association of age with tumor molecular characteristics restricted to variants annotated as oncogenic or likely oncogenic in OncoKB (Data Supplement, Table S2 and Methods) and were conducted separately for each of three tumor types: breast, ovarian, and colorectal. Biological sex was additionally incorporated into post hoc exploratory analyses for CRC but could not be included for the other two tumor types because of the rarity (breast) or absence (ovarian) in men. Age was analyzed as a binary variable defined as AYAs (18-39 years) or non-AYAs (40 or more years), followed by analysis as a continuous variable. Mutations (single nucleotide variant [SNV]/indels) passing initial filters were first examined for their association with age at the gene level, where a gene was considered mutated with at least 1 variant passing the screening criteria (Data Supplement, Table S2). Associations of mutations with binary AYA verus non-AYA status were evaluated using Fisher exact test and with continuous age using logistic regression. Benjamini-Hochberg multiplicity adjusted *P* values (false discovery rate adjusted *P* value [FDRp]) were computed across genes.^35^ Unadjusted *P* values <.05 are reported as trends; FDRp <.10 is considered significant. Similar analyses were performed for genes with amplifications. Fusions were only reported descriptively because of small numbers. SNV/indels were graphically displayed by an oncoprint (ComplexHeatmap Bioconductor package; RRID:SCR_017270) for each of the three tumor groups with annotations for age.

Additional details on the patient cohort, data preprocessing, and statistical analyses are described in Supplemental Methods.

### Ethics Statement

The NCI-MATCH study (ClinicalTrials.gov Identifier: NCT02465060) was approved by the NCI Central Institutional Review Board, the institutional review board of record for all participating institutions. All patients signed a written informed consent document.

## RESULTS

### Ovarian Cancer

A total of 455 ovarian cancers (411 epithelial, 18 stromal, 26 ovarian NOS), representing 21 AYA and 434 non-AYA patients, were included in this analysis (Table 1, Data Supplement, Fig S1). Figure 1A displays an oncoprint of 14 genes that were mutated (SNVs/indels) in six or more patients. *TP53* was the most commonly mutated gene (n = 326/455, 71.6%) in both AYAs (n = 11/21, 52.4%) and non-AYAs (n = 315/434, 72.6%) with a total of 76 patients (five AYAs) for whom the only mutations detected were in *TP53*. Comparisons between AYAs and non-AYAs reveal that *CTNNB1* was mutated nominally more frequently in AYA (n = 2/21, 9.5%) versus non-AYA (n = 4/434, 0.9%) patients (odds ratio [OR] = 0.1 [95% CI, 0.0 to 1.0]) but did not reach statistical significance after multiplicity adjustment (FDRp = 1.0; Table 2 and Data Supplement, Table S3). Of the six ovarian cancers with a *CTNNB1* mutation, three patients, all non-AYA, had a sufficient amount of tumor tissue to confirm endometrioid histologic subtype by histopathology review. Also presented in these tables are results analyzing age as a continuous variable, where *TP53* mutation was significantly associated with older age (Wilcoxon rank-sum FDRp < .001, Fig 1B; 5-year OR, 1.3 [95% CI, 1.1 to 1.4]; FDRp < .001, Fig 1C). No genes mutated only in AYA patients were observed.

**TABLE 2.** Top 20 Genes Ranked by Association of Mutation (single nucleotide variant/indels) With Age in Ovarian, Breast, and Colorectal Cancers

Tumor	Gene	Mutated/Total (%)			Fisher Exact (AYA *v* non-AYA)			Logistic Regression (age in years)		
		AYA	Non-AYA	Overall	*P*	FDRp	R (95% CI)	*P*	FDRp	5-year OR (95% CI)
OVA	***TP53***	11/21 (52.4)	315/434 (72.6)	326/455 (71.6)	.079	1.0	2.4 (0.9 to 6.4)	**3.9e-06**	**.00021**	1.3 (1.1 to 1.4)
	***CTNNB1***	2/21 (9.5)	4/434 (0.9)	6/455 (1.3)	**.027**	1.0	0.1 (0.0 to 1.0)	—	—	—
	***KRAS***	4/21 (19.0)	40/434 (9.2)	44/455 (9.7)	.13	1.0	0.4 (0.1 to 1.9)	**.033**	.45	0.9 (0.8 to 1.0)
	*ATM*	0/18 (0.0)	10/403 (2.5)	10/421 (2.4)	1.0	1.0	—	.060	.54	0.8 (0.6 to 1.0)
	*SMAD4*	1/21 (4.8)	1/433 (0.2)	2/454 (0.4)	.090	1.0	0.0 (0.0 to 3.8)	—	—	—
	*PIK3CA*	1/20 (5.0)	39/430 (9.1)	40/450 (8.9)	1.0	1.0	1.9 (0.3 to 80.7)	.13	.77	0.9 (0.8 to 1.0)
	*MED12*	1/21 (4.8)	2/434 (0.5)	3/455 (0.7)	.13	1.0	0.1 (0.0 to 5.7)	—	—	—
	*FOXL2*	2/21 (9.5)	13/434 (3.0)	15/455 (3.3)	.15	1.0	0.3 (0.1 to 2.9)	.50	.91	1.1 (0.9 to 1.4)
	*NRAS*	1/21 (4.8)	3/434 (0.7)	4/455 (0.9)	.17	1.0	0.1 (0.0 to 7.7)	—	—	—
	*CDKN2A*	1/21 (4.8)	4/434 (0.9)	5/455 (1.1)	.21	1.0	0.2 (0.0 to 9.6)	—	—	—
	*BRCA1*	0/21 (0.0)	38/414 (9.2)	38/435 (8.7)	.24	1.0	—	.38	.91	0.9 (0.8 to 1.1)
	*RB1*	1/20 (5.0)	6/427 (1.4)	7/447 (1.6)	.28	1.0	0.3 (0.0 to 13.1)	—	—	—
	*NF1*	1/21 (4.8)	18/423 (4.3)	19/444 (4.3)	.61	1.0	0.9 (0.1 to 38.9)	.39	.91	0.9 (0.8 to 1.1)
	*BRCA2*	0/21 (0.0)	11/383 (2.9)	11/404 (2.7)	1.0	1.0	—	.44	.91	1.1 (0.9 to 1.5)
	*APC*	0/19 (0.0)	16/394 (4.1)	16/413 (3.9)	1.0	1.0	—	.59	.91	1.1 (0.9 to 1.4)
	*PTEN*	0/21 (0.0)	11/433 (2.5)	11/454 (2.4)	1.0	1.0	—	.81	.94	1.0 (0.8 to 1.4)
	*PPP2R1A*	0/21 (0.0)	9/433 (2.1)	9/454 (2.0)	1.0	1.0	—	—	—	—
	*NOTCH1*	0/19 (0.0)	6/369 (1.6)	6/388 (1.5)	1.0	1.0	—	—	—	—
	*BRAF*	0/21 (0.0)	5/434 (1.2)	5/455 (1.1)	1.0	1.0	—	—	—	—
	*NF2*	0/19 (0.0)	5/426 (1.2)	5/445 (1.1)	1.0	1.0	—	—	—	—
BRC	***CDH1***	0/27 (0.0)	42/526 (8.0)	42/553 (7.6)	.25	1.0	—	**7.8e-05**	**.0041**	1.4 (1.2 to 1.6)
	***PIK3CA***	7/26 (26.9)	187/541 (34.6)	194/567 (34.2)	.53	1.0	1.4 (0.6 to 4.1)	**.00079**	**.021**	1.1 (1.1 to 1.2)
	***NF1***	1/26 (3.8)	27/525 (5.1)	28/551 (5.1)	1.0	1.0	1.4 (0.2 to 57.7)	**.026**	.46	1.2 (1.0 to 1.5)
	***KRAS***	0/27 (0.0)	12/549 (2.2)	12/576 (2.1)	1.0	1.0	—	**.044**	.59	1.3 (1.0 to 1.8)
	***MTOR***	1/27 (3.7)	0/549 (0.0)	1/576 (0.2)	**.047**	1.0	0.0 (0.0 to 1.9)	—	—	—
	*NOTCH1*	0/22 (0.0)	12/476 (2.5)	12/498 (2.4)	1.0	1.0	—	.094	.64	1.3 (1.0 to 1.7)
	*TP53*	15/27 (55.6)	268/544 (49.3)	283/571 (49.6)	.56	1.0	0.8 (0.3 to 1.8)	.16	.64	0.9 (0.9 to 1.0)
	*ESR1*	3/27 (11.1)	127/547 (23.2)	130/574 (22.6)	.16	1.0	2.4 (0.7 to 12.7)	.77	.87	1.0 (0.9 to 1.1)
	*ERBB2*	0/26 (0.0)	22/537 (4.1)	22/563 (3.9)	.62	1.0	—	.17	.64	1.1 (0.9 to 1.4)
	*VHL*	1/27 (3.7)	3/545 (0.6)	4/572 (0.7)	.18	1.0	0.1 (0.0 to 7.8)	—	—	—
	*ATM*	2/27 (7.4)	14/498 (2.8)	16/525 (3.0)	.20	1.0	0.4 (0.1 to 3.5)	.58	.81	0.9 (0.8 to 1.2)
	*HRAS*	1/27 (3.7)	5/548 (0.9)	6/575 (1.0)	.25	1.0	0.2 (0.0 to 11.8)	—	—	—
	*APC*	0/24 (0.0)	14/497 (2.8)	14/521 (2.7)	1.0	1.0	—	.31	.78	1.1 (0.9 to 1.5)
	*AKT1*	2/27 (7.4)	22/549 (4.0)	24/576 (4.2)	.31	1.0	0.5 (0.1 to 4.8)	.57	.81	1.1 (0.9 to 1.3)
	*PTEN*	1/27 (3.7)	37/543 (6.8)	38/570 (6.7)	1.0	1.0	1.9 (0.3 to 80.0)	.35	.81	1.1 (0.9 to 1.2)
	*CHEK2*	1/27 (3.7)	8/549 (1.5)	9/576 (1.6)	.35	1.0	0.4 (0.0 to 17.7)	—	—	—
	*BRCA1*	0/24 (0.0)	13/510 (2.5)	13/534 (2.4)	1.0	1.0	—	.46	.81	1.1 (0.9 to 1.4)
	*TSC2*	0/25 (0.0)	12/501 (2.4)	12/526 (2.3)	1.0	1.0	—	.50	.81	1.1 (0.8 to 1.4)
	*GATA3*	1/26 (3.8)	58/537 (10.8)	59/563 (10.5)	.51	1.0	3.0 (0.5 to 126.3)	.53	.81	1.0 (0.9 to 1.1)
	*RB1*	1/27 (3.7)	21/537 (3.9)	22/564 (3.9)	1.0	1.0	1.1 (0.2 to 45.4)	.79	.87	1.0 (0.8 to 1.2)
CRC	***NOTCH1***	0/36 (0.0)	10/612 (1.6)	10/648 (1.5)	1.0	1.0	—	**.0031**	.17	1.7 (1.2 to 2.4)
	***BRAF***	1/43 (2.3)	56/716 (7.8)	57/759 (7.5)	.24	1.0	3.6 (0.6 to 146.5)	**.0058**	.17	1.2 (1.1 to 1.3)
	***PTEN***	0/43 (0.0)	26/707 (3.7)	26/750 (3.5)	.39	1.0	—	**.016**	.25	1.2 (1.0 to 1.5)
	***MET***	0/43 (0.0)	11/706 (1.6)	11/749 (1.5)	1.0	1.0	—	**.020**	.25	1.4 (1.1 to 1.9)
	***SMAD4***	6/41 (14.6)	60/692 (8.7)	66/733 (9.0)	.25	1.0	0.6 (0.2 to 1.7)	**.036**	.35	0.9 (0.8 to 1.0)
	***APC***	26/39 (66.7)	556/688 (80.8)	582/727 (80.1)	**.039**	1.0	2.1 (1.0 to 4.4)	.81	.91	1.0 (0.9 to 1.1)
	*NF1*	2/43 (4.7)	14/680 (2.1)	16/723 (2.2)	.25	1.0	0.4 (0.1 to 4.0)	.091	.53	0.8 (0.7 to 1.0)
	*GNAS*	1/43 (2.3)	9/714 (1.3)	10/757 (1.3)	.44	1.0	0.5 (0.1 to 24.1)	.14	.54	0.8 (0.6 to 1.1)
	*TP53*	27/42 (64.3)	538/712 (75.6)	565/754 (74.9)	.14	1.0	1.7 (0.8 to 3.4)	.51	.75	1.0 (0.9 to 1.0)
	*PIK3R1*	1/43 (2.3)	20/701 (2.9)	21/744 (2.8)	1.0	1.0	1.2 (0.2 to 52.3)	.15	.54	1.2 (1.0 to 1.4)
	*CDKN2A*	1/43 (2.3)	2/706 (0.3)	3/749 (0.4)	.16	1.0	0.1 (0.0 to 7.2)	—	—	—
	*PTCH1*	1/42 (2.4)	2/650 (0.3)	3/692 (0.4)	.17	1.0	0.1 (0.0 to 7.6)	—	—	—
	*ERBB2*	2/43 (4.7)	12/699 (1.7)	14/742 (1.9)	.19	1.0	0.4 (0.1 to 3.4)	.86	.91	1.0 (0.8 to 1.3)
	*CTNNB1*	1/43 (2.3)	3/713 (0.4)	4/756 (0.5)	.21	1.0	0.2 (0.0 to 9.5)	—	—	—
	*KDR*	1/43 (2.3)	3/705 (0.4)	4/748 (0.5)	.21	1.0	0.2 (0.0 to 9.6)	—	—	—
	*FBXW7*	5/42 (11.9)	49/694 (7.1)	54/736 (7.3)	.22	1.0	0.6 (0.2 to 1.9)	.45	.75	1.0 (0.9 to 1.2)
	*PIK3CA*	11/43 (25.6)	128/710 (18.0)	139/753 (18.5)	.22	1.0	0.6 (0.3 to 1.4)	.23	.57	1.1 (1.0 to 1.1)
	*MAP2K1*	1/43 (2.3)	4/715 (0.6)	5/758 (0.7)	.25	1.0	0.2 (0.0 to 11.9)	—	—	—
	*CDH1*	1/42 (2.4)	5/702 (0.7)	6/744 (0.8)	.30	1.0	0.3 (0.0 to 14.2)	—	—	—
	*EGFR*	1/42 (2.4)	6/703 (0.9)	7/745 (0.9)	.33	1.0	0.4 (0.0 to 16.6)	—	—	—

NOTE. Genes that were mutated in ovarian (OVA), BRC, CRC ordered from smallest to largest of minimum of the Fisher exact and logistic regression unadjusted *P* values. For genes with a minimum *P* value of 1, genes are ordered first by total no. of observed mutations. For the Fisher exact test (comparing proportion mutated between AYA and non-AYA groups), OR >1 means that mutations are more frequent in the non-AYA group. ORs were not presented when undefined due to division by 0. For the logistic regression (association of gene mutation with age as a continuous variable), ORs greater than 1 and less than 1 represent the association of mutation in the gene with older and younger age, respectively. Logistic regression was not performed for genes that were mutated in <10 patients. FDRp is a *P* value adjusted for multiple comparisons using the Benjamini-Hochberg method (Supplemental Methods). Genes with unadjusted *P* value <.05 or FDRp < .1 are in bold. The columns under Mutated/Total (%) show number of patients with the gene having at least 1 mutation, total number of patients analyzed, and percent of patients with the gene mutated for each age group and overall. At a patient level, any mutation with read depth of <250, or variant read depth <25, or with inconsistent/unknown oncogenicity was filtered out and that led to different total number of patients analyzed for each gene.

Abbreviations: AYA, adolescent and young adult; BRC, breast cancer; CRC, colorectal cancer; FDRp, false discovery rate‑adjusted *P* value; OR, odds ratio; OVA, ovarian cancer.

Amplifications of 24 genes were observed across 127 patients (five AYAs, 123 non-AYAs). No statistically significant differences were observed in comparing amplifications in AYAs and non-AYAs (Data Supplement, Table S4). When analyzing age as a continuous variable (Data Supplement, Table S4), both *CCNE1* and *MYCL* amplification exhibited a trend for association with older age (5-year OR, 1.3 [95% CI, 1.1 to 1.6]; FDRp = .06 and 5-year OR, 1.4 [95% CI, 1.0 to 1.9]; FDRp = .44, respectively), while only *CCNE1* amplifications remained significant following multiplicity adjustment (Wilcoxon rank-sum FDRp = .009, Fig 1D; FDRp = .06, Fig 1E). Fusion events are presented in the Data Supplement (Table S5), with none occurring in AYA patients.

Sensitivity analyses were conducted in ovarian epithelial (n = 411) and stromal (n = 18) cancers separately (Data Supplement, Table S6). *TP53* was significantly associated with older age in ovarian epithelial cancer (FDRp < .001). *FOXL2* (402C>G, C134W) was only detected in stromal samples and was the most frequent (78%) mutation with a trend for association with older age (unadjusted *P* value = .048).

### Breast Cancer

**FIG 2.**
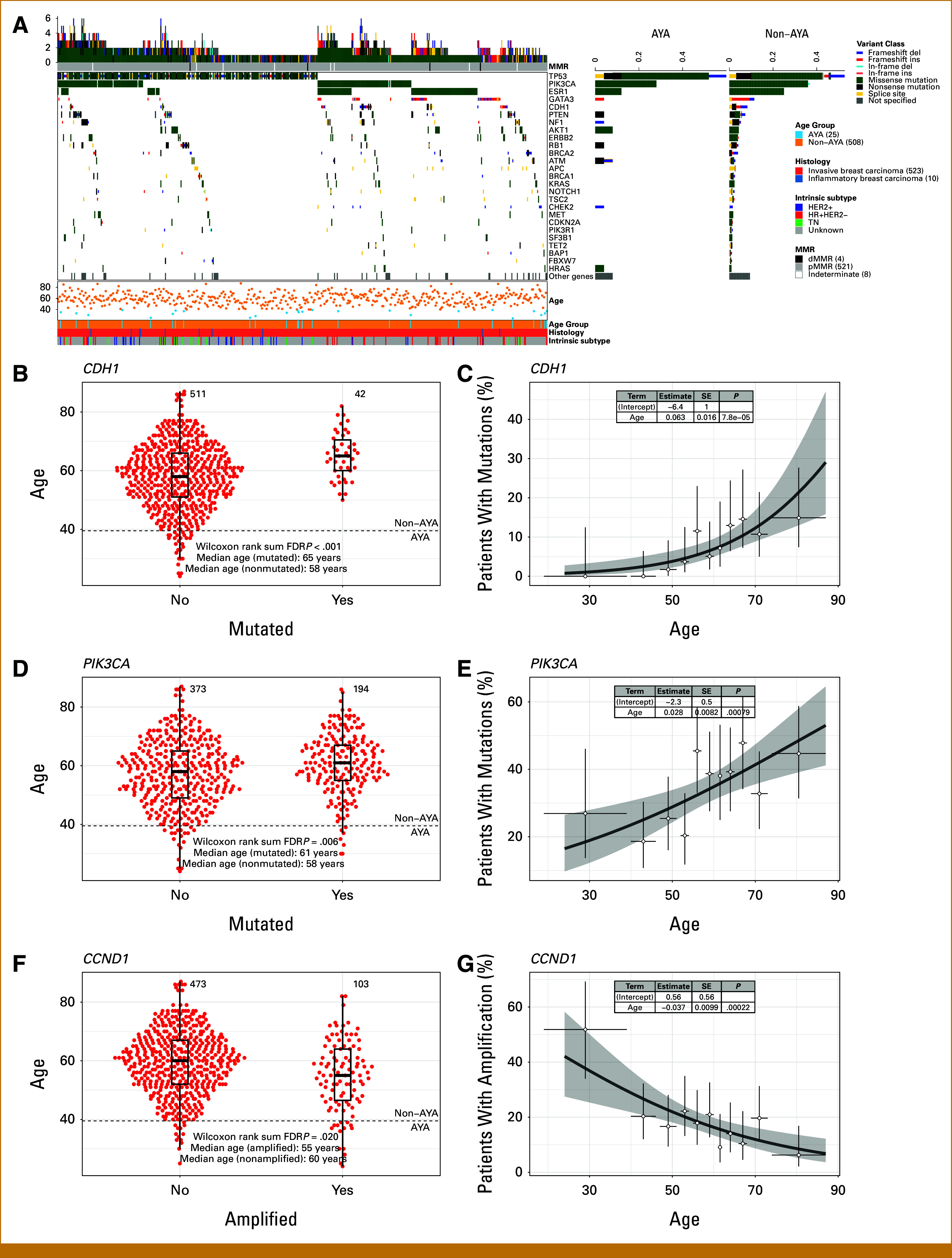
(A) The genomic landscape (SNV/indels) of 533 (25 AYA) patients with breast cancer screened on the NCI-MATCH trial. Only patients with at least 1 mutation are shown, excluding 43 patients. Twenty-six genes were mutated in more than five patients. See captions for Figure 1A for other details. (B-G) Association of age with selected alterations in breast cancer: (B, C) *CDH1* mutation; (D, E) *PIK3CA* mutation; (F, G) *CCND1* amplification. See captions for Figures 1B and 1C for other details. AYA, adolescent and young adult; dMMR, deficient mismatch repair; FDRp, false discovery rate‑adjusted *P* value; HER2, human epidermal growth factor receptor 2; HR, hormone receptor; pMMR, proficient mismatch repair; SNV, single nucleotide variant; TN, triple-negative.

A total of 576 BRCs (566 invasive carcinoma, 10 inflammatory), representing 27 AYA and 549 non-AYA patient cases, were included in this analysis (Table 1, Data Supplement, Fig S1). Figure 2A displays an oncoprint of 26 genes that were mutated (SNVs/indels) in six or more patients. *TP53* was the most commonly mutated gene (n = 283/571, 49.6%) in both AYA (n = 15/27, 55.6%) and non-AYA (n = 268/544, 49.3%) patients. Comparisons between AYA and non-AYA patients reveal no genes for which the proportion of AYA patients with mutation differed significantly from that in non-AYA patients (Table 2 and Data Supplement, Table S3). Also presented in Table 2 are results analyzing age as a continuous variable, demonstrating that mutations were significantly associated with older age for *CDH1* (Wilcoxon rank-sum FDRp < .001, Fig 2B; 5-year OR, 1.4 [95% CI, 1.2 to 1.6]; FDRp = .004, Fig 2C) and *PIK3CA* (Wilcoxon rank-sum FDRp = .006, Fig 2D; 5-year OR, 1.1 [95% CI, 1.1 to 1.2], FDRp = .02, Fig 2E). Additionally, results showed a trend toward mutations in *NF1* and *KRAS* being associated with older age, but these findings were not statistically significant after multiplicity adjustment (FDRp = .46 and .59, respectively). One gene, *MTOR* (C1483F variant), was mutated in a single AYA patient but not in any non-AYA patient.

Twenty-eight genes had an amplification detected among 301 patients (20 AYA, 281 non-AYA). Comparisons between AYA and non-AYA patients reveal that *CCND1* amplification (11q13.3) was significantly more frequent in AYA compared with non-AYA patients (OR, 0.2 [95% CI, 0.1 to 0.4]), FDRp = .001 (Data Supplement, Table S4). When analyzing age as a continuous variable, *CCND1* and *MYC* amplification showed a trend toward association with younger age (unadjusted *P* value <.05), although only the association with *CCND1* amplification (Wilcoxon rank-sum FDRp = .02, Fig 2F; 5-year OR, 0.8 [95% CI, 0.8 to 0.9], FDRp = .006, Fig 2G) remained significant following multiplicity adjustment. Fusion events are described in Data Supplement (Table S5), with none occurring in AYA patients.

Sensitivity analyses were conducted to assess the association of age with BRC subtype (ER/PgR/HER2) grouping, and no genes exhibited a trend for an interaction effect between age and subtype (Supplemental Results).

### Colorectal Cancer

**FIG 3.**
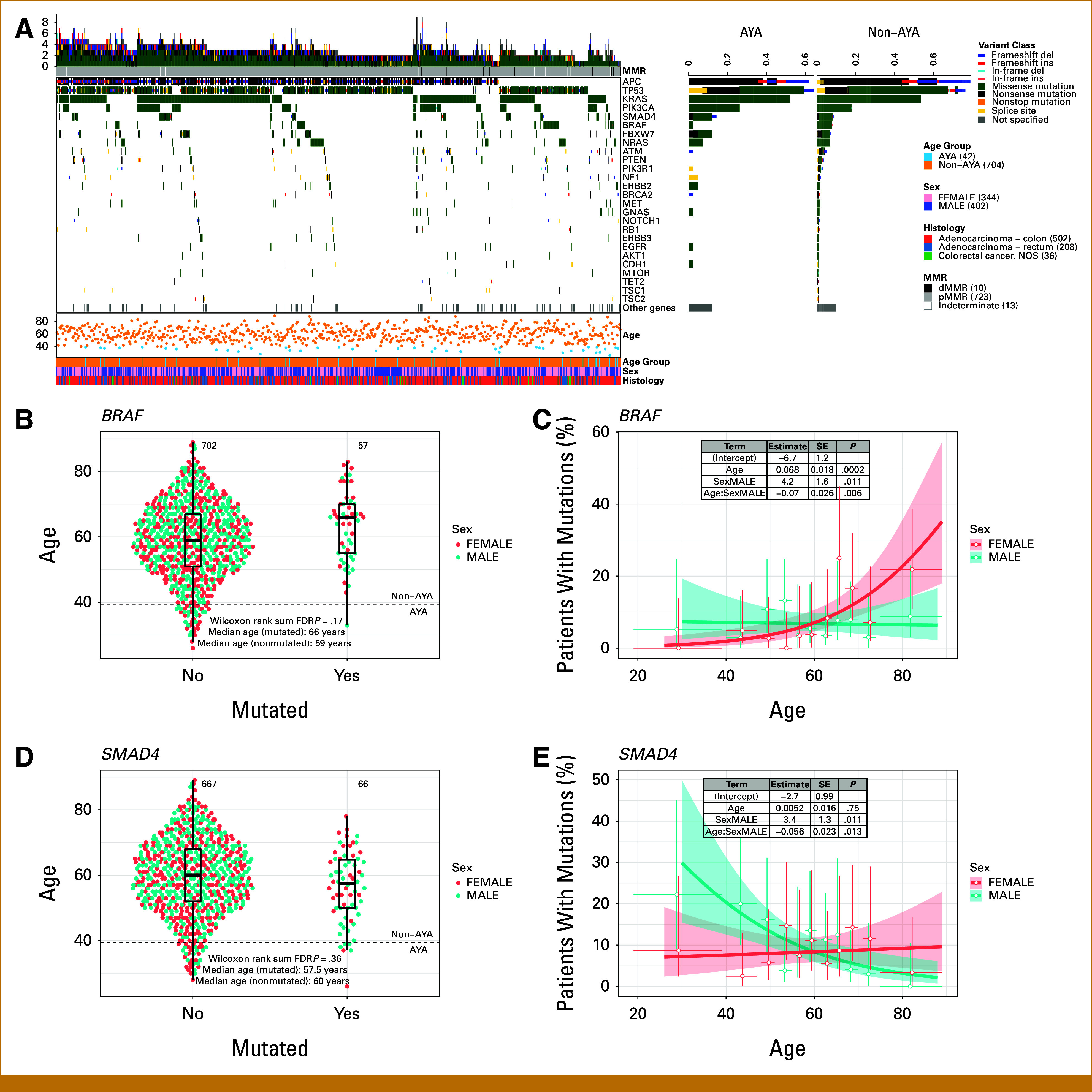
(A) The genomic landscape (SNV/indels) of 746 (42 AYA) patients with colorectal cancer screened on the NCI-MATCH trial. Only patients with at least 1 mutation are shown, excluding 13 patients. Twenty-six genes were mutated in more than five patients. See captions for Figure 1A for other details. (B-E) Association of age with selected alterations in colorectal cancer: (B, C) *BRAF* mutation; (D, E) *SMAD4* mutation. See captions for Figures 1B and 1C for other details. For C and E, for each age bin, the center is represented by a dot with a small shift to better visualize male and female patients. AYA, adolescent and young adult; dMMR, deficient mismatch repair; FDRp, false discovery rate‑adjusted *P* value; NOS, not otherwise specified; pMMR, proficient mismatch repair; SNV, single nucleotide variant.

A total of 759 colorectal cancers (512 adenocarcinoma colon, 210 adenocarcinoma rectum, 37 colorectal NOS), representing 43 AYA and 716 non-AYA patients, were included in this analysis (Table 1, Data Supplement, Fig S1). Figure 3A displays an oncoprint of 26 genes that were mutated in six or more patients. *APC* was the most commonly mutated gene (n = 582/727, 80.1%) in both AYA (n = 26/39, 66.7%) and non-AYA (n = 556/688, 80.8%) patients. Comparisons between AYAs and non-AYAs (Table 2 and Data Supplement, Table S3) reveal that *APC* was mutated nominally more frequently in non-AYA versus AYA patients, but this result was not statistically significant after multiplicity adjustment (OR, 2.1 [95% CI, 1.0 to 4.4], unadjusted *P* value = .04, FDRp = 1.0). Although *NOTCH1*, *BRAF*, *PTEN*, and *MET* mutations were observed nominally associated with older age when analyzing age as a continuous variable (unadjusted *P* values: .003, .006, .016, and .02, respectively), none were significant after multiplicity adjustment. *SMAD4* was observed nominally associated with younger age (unadjusted *P* value = .03) but was not statistically significant after multiplicity adjustment (FDRp = .35). No genes mutated only in AYA patients were observed.

No statistically significant associations of gene amplifications with age were found, either comparing AYA and non-AYA patients or considering age as a continuous variable (Data Supplement, Table S4). Fusion events are summarized in the Data Supplement, Table S5, with only 1 occurring in AYA patients.

The Data Supplement (Table S7) presents results for three genes with a trend for an interaction effect (unadjusted *P* value <.05) between age and sex. For these three genes (*BRAF*, *SMAD4*, and *ATM*), exploratory subgroup analyses were conducted to assess the association of mutation with age in men (n = 411) and women (n = 348) separately. *BRAF* was significantly associated with older age in women (5-year OR, 1.4 [95% CI, 1.2 to 1.7], FDRp = .01) and is displayed in Figures 3B and 3C. *SMAD4* was significantly associated with younger age in men (5-year OR = 0.8, 95% CI: 0.7-0.9, FDRp = .06) and is displayed in Figures 3D and 3E.

Sensitivity analyses were conducted in colon (n = 512) and rectal (n = 210) cancers separately and revealed no significant associations by FDRp in either group (Data Supplement, Table S8).

### Other Analyses

Location and type of variant in genes with mutation in at least 10% of patients in one of the three tumor types and significantly associated with age (logistic regression unadjusted *P* value <.05; *TP53*, *KRAS*, and *PIK3CA*) were visually compared using lollipop plots (Data Supplement, Figs S2-S4). Analysis of associations of age with tier 1 and 2 variants revealed significant associations for *PIK3CA* p.H10479R in older patients with BRC and *BRAF* p.V600E in older patients with colorectal cancer (Table 3, Data Supplement, Table S9).

**TABLE 3.** Analysis of Tier 1 Variants Within Genes in Table 2 Exhibiting Significant Association of Mutation With Age

Tumor	Variant Information					Mutated/Total (%)			Fisher Exact (AYA *v* Non-AYA)		Logistic Regression (age in years)		
	Gene	Genomic Location	Genomic Change	AA Change	Variant Classification	AYA	Non-AYA	Overall	*P*	OR (95% CI)	*P*	5-year OR (95% CI)	Age in Mutated: Median (range)
BRC	***PIK3CA***	chr3:178952085	A>G	p.H1047R	Missense mutation	5/27 (18.5)	68/549 (12.4)	73/576 (12.7)	.37	0.6 (0.2 to 2.2)	**.019**	1.1 (1.0 to 1.3)	63 (30-77)
	*PIK3CA*	chr3:178936091	G>A	p.E545K	Missense mutation	1/27 (3.7)	40/549 (7.3)	41/576 (7.1)	.71	2.0 (0.3 to 85.8)	.43	1.1 (0.9 to 1.2)	60 (38-77)
	*PIK3CA*	chr3:178936082	G>A	p.E542K	Missense mutation	1/27 (3.7)	29/549 (5.3)	30/576 (5.2)	1.0	1.4 (0.2 to 61.5)	.44	1.1 (0.9 to 1.3)	61 (37-85)
	*PIK3CA*	chr3:178952085	A>T	p.H1047L	Missense mutation	0/27 (0.0)	13/549 (2.4)	13/576 (2.3)	1.0	—	.53	0.9 (0.7 to 1.2)	57 (46-67)
	*PIK3CA*	chr3:178921553	T>A	p.N345K	Missense mutation	0/27 (0.0)	10/549 (1.8)	10/576 (1.7)	1.0	—	.60	1.1 (0.8 to 1.4)	57.5 (46-77)
	*PIK3CA*	chr3:178916876	G>A	p.R88Q	Missense mutation	0/27 (0.0)	3/549 (0.5)	3/576 (0.5)	1.0	—	—	—	67 (63-68)
	*PIK3CA*	chr3:178927980	T>C	p.C420R	Missense mutation	0/26 (0.0)	3/548 (0.5)	3/574 (0.5)	1.0	—	—	—	60 (55-64)
	*PIK3CA*	chr3:178952090	G>C	p.G1049R	Missense mutation	0/27 (0.0)	3/549 (0.5)	3/576 (0.5)	1.0	—	—	—	59 (58-63)
	*PIK3CA*	chr3:178916728	G>A	p.E39K	Missense mutation	0/27 (0.0)	2/549 (0.4)	2/576 (0.3)	1.0	—	—	—	(73-76)
	*PIK3CA*	chr3:178917478	G>A	p.G118D	Missense mutation	0/27 (0.0)	2/549 (0.4)	2/576 (0.3)	1.0	—	—	—	(46-55)
	*PIK3CA*	chr3:178936082	G>C	p.E542Q	Missense mutation	0/27 (0.0)	2/549 (0.4)	2/576 (0.3)	1.0	—	—	—	(74-76)
	*PIK3CA*	chr3:178936091	G>C	p.E545Q	Missense mutation	0/27 (0.0)	2/549 (0.4)	2/576 (0.3)	1.0	—	—	—	(66-79)
	*PIK3CA*	chr3:178936095	A>G	p.Q546R	Missense mutation	0/27 (0.0)	2/549 (0.4)	2/576 (0.3)	1.0	—	—	—	(41-59)
	*PIK3CA*	chr3:178952077	T>A	p.N1044K	Missense mutation	0/27 (0.0)	2/549 (0.4)	2/576 (0.3)	1.0	—	—	—	(42-66)
	*PIK3CA*	chr3:178916924	CAG>-	p.P104_V105delinsL	In frame Del	0/27 (0.0)	1/549 (0.2)	1/576 (0.2)	1.0	—	—	—	77
	*PIK3CA*	chr3:178916941	GAAAAGATC>-	p.E110_I112del	In frame Del	0/27 (0.0)	1/549 (0.2)	1/576 (0.2)	1.0	—	—	—	69
	*PIK3CA*	chr3:178916944	A>G	p.K111E	Missense mutation	0/27 (0.0)	1/549 (0.2)	1/576 (0.2)	1.0	—	—	—	68
	*PIK3CA*	chr3:178916944	AAG>-	p.K111del	In frame Del	0/27 (0.0)	1/549 (0.2)	1/576 (0.2)	1.0	—	—	—	86
	*PIK3CA*	chr3:178916946	G>C	p.K111N	Missense mutation	0/27 (0.0)	1/549 (0.2)	1/576 (0.2)	1.0	—	—	—	58
	*PIK3CA*	chr3:178917496	C>T	p.P124L	Missense mutation	0/27 (0.0)	1/549 (0.2)	1/576 (0.2)	1.0	—	—	—	61
	*PIK3CA*	chr3:178928079	GAAGAT>-	p.E453_D454del	In frame Del	0/27 (0.0)	1/549 (0.2)	1/576 (0.2)	1.0	—	—	—	51
	*PIK3CA*	chr3:178936074	C>G	p.P539R	Missense mutation	0/27 (0.0)	1/549 (0.2)	1/576 (0.2)	1.0	—	—	—	61
	*PIK3CA*	chr3:178936092	A>C	p.E545A	Missense mutation	0/27 (0.0)	1/549 (0.2)	1/576 (0.2)	1.0	—	—	—	61
	*PIK3CA*	chr3:178936094	C>A	p.Q546K	Missense mutation	0/27 (0.0)	1/549 (0.2)	1/576 (0.2)	1.0	—	—	—	70
	*PIK3CA*	chr3:178936095	A>C	p.Q546P	Missense mutation	0/27 (0.0)	1/549 (0.2)	1/576 (0.2)	1.0	—	—	—	67
	*PIK3CA*	chr3:178936104	A>G	p.D549G	Missense mutation	0/27 (0.0)	1/549 (0.2)	1/576 (0.2)	1.0	—	—	—	48
	*PIK3CA*	chr3:178947827	G>T	p.C901F	Missense mutation	0/27 (0.0)	1/549 (0.2)	1/576 (0.2)	1.0	—	—	—	59
	*PIK3CA*	chr3:178948043	G>A	p.D939N	Missense mutation	0/27 (0.0)	1/549 (0.2)	1/576 (0.2)	1.0	—	—	—	67
	*PIK3CA*	chr3:178948044	A>G	p.D939G	Missense mutation	0/27 (0.0)	1/549 (0.2)	1/576 (0.2)	1.0	—	—	—	62
	*PIK3CA*	chr3:178952006	T>C	p.Y1021H	Missense mutation	0/27 (0.0)	1/549 (0.2)	1/576 (0.2)	1.0	—	—	—	66
	*PIK3CA*	chr3:178952018	A>G	p.T1025A	Missense mutation	0/27 (0.0)	1/549 (0.2)	1/576 (0.2)	1.0	—	—	—	72
	*PIK3CA*	chr3:178952084	C>T	p.H1047Y	Missense mutation	0/27 (0.0)	1/549 (0.2)	1/576 (0.2)	1.0	—	—	—	57
CRC	***BRAF***	chr7:140453136	A>T	p.V600E	Missense mutation	1/43 (2.3)	36/716 (5.0)	37/759 (4.9)	.72	2.2 (0.4 to 92.3)	**.0013**	1.3 (1.1 to 1.5)	66 (33-82)

NOTE. Variants are ordered by total no. of observed mutations, separately by BRC and CRC. No tier 1 variants were detected in ovarian cancer. For the Fisher exact test (comparing proportion mutated between AYA and non-AYA groups), OR >1 means that mutations are more frequent in the non-AYA group. ORs were not presented when undefined due to division by 0. For the logistic regression (association of gene mutation with age as a continuous variable), ORs greater than 1 and less than 1 represent association of mutation with older and younger age, respectively. Logistic regression was not performed for variants that were mutated in < 10 patients. Variants with *P* value < .05 are in bold. See Supplemental Methods for details regarding mapping of OncoKB levels to ASCO/AMP/CAP tier classification.

Abbreviations: AYA, adolescent and young adult; BRC, breast cancer; CRC, colorectal cancer; OR, odds ratio.

Associations of age with altered DNA repair pathway and deficient mismatch repair (dMMR) were evaluated. Altered DNA repair pathway showed significant association with younger age in BRC and older age in ovarian cancer, mostly driven by *TP53* (Data Supplement, Fig S5). No significant association was found with age for dMMR (Data Supplement, Fig S6).

## DISCUSSION

Results reported in this article, representing 91 AYA and 1,699 non-AYA patients with breast, ovarian, or colorectal cancer, show trends in prevalence of certain DNA alterations according to age that may have potential therapeutic implications, as reflected in tier 1 variants presented in Table 3, or point toward future research opportunities.

Molecular heterogeneity of ovarian cancer observed in this heavily pretreated cohort recapitulates the heterogeneity previously described at the time of initial diagnosis in other cohorts.^5,8,9^ Expected *TP53* mutations were observed frequently, as were alterations in homologous recombination genes and *KRAS*.^5^ Only *TP53* reached statistical significance after FDR adjustment for association with age, as a continuous variable, but trends (not statistically significant) were observed for *CTNNB1* and *KRAS*. *TP53* mutations are known to accumulate with age, consistent with findings in this study,^5^ although the association was not driven specifically by differences between AYA and non-AYA subgroups. The role of *CCNE1* amplification in younger women is unclear but has been proposed as a potential indicator of platinum resistance,^36^ and our data suggested an association with TP53 mutation (data not shown). The preponderance of the *FOXL2* C134W variant in ovarian stromal cancers like granulosa cell tumors is well described, although the observed trend with older age warrants further study.^37^

HER2+, hormone receptor–positive (HR+)/HER2−, and ER−/PgR–/HER2− BRC subtypes are now well integrated into clinical practice, but within these subtypes heterogeneity of other clinicopathologic features and outcomes remains. HER2+ and ER−/PgR–/HER2− (predominantly basal subtype) are known to be more highly represented in younger patients, likely contributing to worse outcomes.^18^ However, in HR+ tumors, prognosis is worse for women younger than 40 years compared with older, even after accounting for standard pathologic features.^20,38,39^ In this study, no genes were identified for which prevalence of mutation differed by age dichotomized as AYAs versus non-AYAs, but significant associations were identified with age as a continuous variable. Mutations in *PIK3CA* and *CDH1* (E-cadherin) were observed to be less common in younger women, consistent with a prior study.^21^ Considering that lack of E-cadherin expression (likely due to mutation) is characteristic of invasive lobular carcinoma, findings here are consistent with the lobular subtype tending to be diagnosed at older age.^19^ A preponderance of *TP53* mutations in younger patients with BRC was expected but not observed, possibly because of heavy pretreatment of this study cohort.

BRC gene amplifications revealed associations with age. *CCND1* amplification was significantly more common in younger women (continuous age); to our knowledge, this is a novel finding. Chi et al evaluated differences in CNAs between women younger than 45 years and older women with BRC in both a discovery and validation set from the METABRIC (Molecular Taxonomy of Breast Cancer International Consortium) database.^40^ They validated 51 recurrent copy number gain regions in younger women, but none of these contained *CCND1* amplification. Another study evaluated high-frequency CNAs in 259 young women (younger than 55 years) and did not find any amplification of 11q13, the location of *CCND1*.^41^

A combination of whole-exome and targeted panel sequencing studies have revealed possible unique mutational changes in the tumor genomes of AYA patients with CRC,^29-31^ suggesting that colorectal cancers in younger patients may be driven by a different biology.^42,43^ In these studies, including some that used pretreatment samples, several genes exhibited significant mutational frequency differences between AYA and older patients including *POLE*, *FBXW7*, *MYCBP2*, *BRCA2*, *PHLPP1*, *TOPORS*, and *ATR*, of which only *BRCA2* and *FBXW7* were represented on the NCI-MATCH targeted panel. Statistically significant mutational frequency differences observed in our study for colorectal cancer were *SMAD4* mutation in younger men and *BRAF* mutation in older women. *SMAD4* encodes a component of the TGF-beta signaling pathway and has been shown to act as a tumor suppressor in epithelial cells and is associated with juvenile polyposis syndrome.^44^ *BRAF* encodes a protein belonging to the RAF family of serine/threonine protein kinases and is associated with numerous human cancers including colorectal cancer.^45^ *BRAF* mutations have been associated with older age and female sex, consistent with findings in this study, and with worse prognosis in metastatic colorectal cancer^46^ and at a frequency similar to that reported in this study.^47^ Observation of a sharp increase in rate of *BRAF* mutation in women but not men, beginning around age 60 (sex-by-age interaction), is consistent with a mechanistic hypothesis that estrogen may be protective against microsatellite instability (MSI),^32^ a potential consequence of BRAF V600E mutation.^48^ Through this mechanism, *BRAF*-mutated tumors might be less likely suppressed (more prevalent) in older (postmenopausal) women with diminished estrogen levels.

Some study limitations must be acknowledged. Genomic data used in these analyses were generated using the NCI-MATCH assay (v2), which is a 143-gene panel containing most known common oncogenic drivers of tumor growth. A more comprehensive analysis (such as exome sequencing and RNAseq) could potentially provide additional insights about associations with complex biomarkers such as tumor mutational burden, MSI, and homologous recombination deficiency, which cannot be interrogated reliably on panels the size of the NCI-MATCH assay. Additionally, the assay did not use patient-matched normal germline DNA, thereby hindering identification of germline mutations, which could be more common in AYAs with cancers. Mutational profiles may also evolve over time in response to therapy such that the associations identified in this study of heavily pretreated patients could be different from those observed in a newly diagnosed patient population. Detailed pathologic classifications (beyond MedDRA v10.0 disease terms), disease-specific standard clinicopathologic variables, treatment history, family history, and epidemiologic data were not comprehensively collected in NCI-MATCH, limiting the ability to compare results with prior studies of more restricted patient cohorts, including those of newly diagnosed patients before treatment. The treatment-actionable focus of the NCI-MATCH gene panel also limits the ability to glean insights into cancer causation and risk or opportunities for prevention. Further discussion of limitations and potential biases is provided in the Supplement Limitations.

Despite the NCI-MATCH cohort's large size, the number of AYAs was still modest and eligibility excluded patients younger than 18 years. Together with the low prevalence of many genomic alterations, statistical power to detect some associations would have been low (Data Supplement, Table S10).

In conclusion, a wealth of information can be gleaned from a national-scale trial like NCI-MATCH that collected tumor molecular profiles generated from a standardized assay in thousands of patients with recurrent/refractory disease accrued from a wide geographic region, reflecting many tumor types and clinical and demographic characteristics. Future analyses of the NCI-MATCH screening cohort will examine associations of tumor profiles with additional clinical and demographic factors.

## Data Availability

A data sharing statement provided by the authors is available with this article at DOI https://doi.org/10.1200/PO-25-01183.
